# Valve-in-Valve Transcatheter Aortic Valve Implantation Using Fluoroscopic Guidance for Late Failure of the Bioprosthetic Bentall Conduit BioValsalva™ Vascutek

**DOI:** 10.7759/cureus.12006

**Published:** 2020-12-10

**Authors:** Pedro Teixeira, Wilson Ferreira, Gustavo Pires-Morais, Alberto Rodrigues, Pedro Braga

**Affiliations:** 1 Cardiology, Centro Hospitalar de Vila Nova de Gaia / Espinho, Vila Nova de Gaia, PRT; 2 Cardiology, Centro Hospitalar de Vila Nova de Gaia / Espinho, Vila Nova De Gaia, PRT

**Keywords:** transcatheter aortic valve implantation, valve-in-valve, bio-prosthetic conduit failure

## Abstract

We describe one of the first-in-human cases of valve-in-valve transcatheter aortic valve implantation (TAVI) using fluoroscopic-only guidance for the treatment of late failure of a bio-prosthetic Bentall conduit, the BioValsalva™ Vascutek (Vascutek Terumo, Renfrewshire, Scotland), using a self-expandable heart valve prosthesis (CoreValve™^ ^Evolut™ R, Medtronic, Dublin Ireland).

## Introduction

Over the past decade, transcatheter aortic valve implantation (TAVI) has emerged as a valid and safe treatment option for severe symptomatic aortic stenosis, from prohibitive surgical risk patients in the early days to those at low-to-intermediate risk in current practice [1-6].

Advances in device technology, as well as growing operators’ skills, were remarkable in this short time window. This set the stage for a fantastic expansion of the procedure indications towards increasingly challenging clinical scenarios. Valve-in-valve TAVI has become a safe and reproducible procedure for patients with severe bioprosthetic valve dysfunction deemed to be at high risk for re-operation.

Pure (native or prosthetic) valve regurgitation is still a grey zone, and evidence in this challenging subset is much needed. These patients typically exhibit less annular and/or leaflet calcifications, implying technical difficulties in adequate transcatheter aortic valve anchoring. Furthermore, non-calcified native aortic valves and non-radiopaque bioprosthesis both challenge correct positioning under fluoroscopic guidance.

We describe one of the first in-human cases of valve-in-valve TAVI using fluoroscopic-only guidance for the treatment of late failure of a bio-prosthetic Bentall conduit, the BioValsalva™ Vascutek (Vascutek Terumo, Renfrewshire, Scotland), using a self-expandable heart valve prosthesis (CoreValve™ Evolut™ R, Medtronic, Dublin Ireland).

## Case presentation

The patient was a 65-year-old woman with no known cardiovascular risk factors. Relevant past medical history included epilepsy and primary biliary cirrhosis. She was submitted to a Bentall surgical procedure, with implantation of a 25mm porcine bioprosthetic valved conduit (BioValsalva™ Vascutek) in 2008, following documentation of bicuspid aortic valve with severe regurgitation and a dilated ascending aorta.

She was chronically medicated with venlafaxine, levetiracetam, pantoprazole, aspirin, furosemide, and bisoprolol.

The patient presented with new-onset heart failure symptoms twelve-years after the Bentall procedure, New York Heart Association (NYHA) III functional class, and severe transprosthetic aortic regurgitation was diagnosed. She was refused for re-do cardiac surgery considering high surgical risk - the calculated EuroScore II was 18.52%, and the Society of Thoracic Surgeons (STS) score for mortality was 8.60%.

Procedural planning

**Figure 1:**
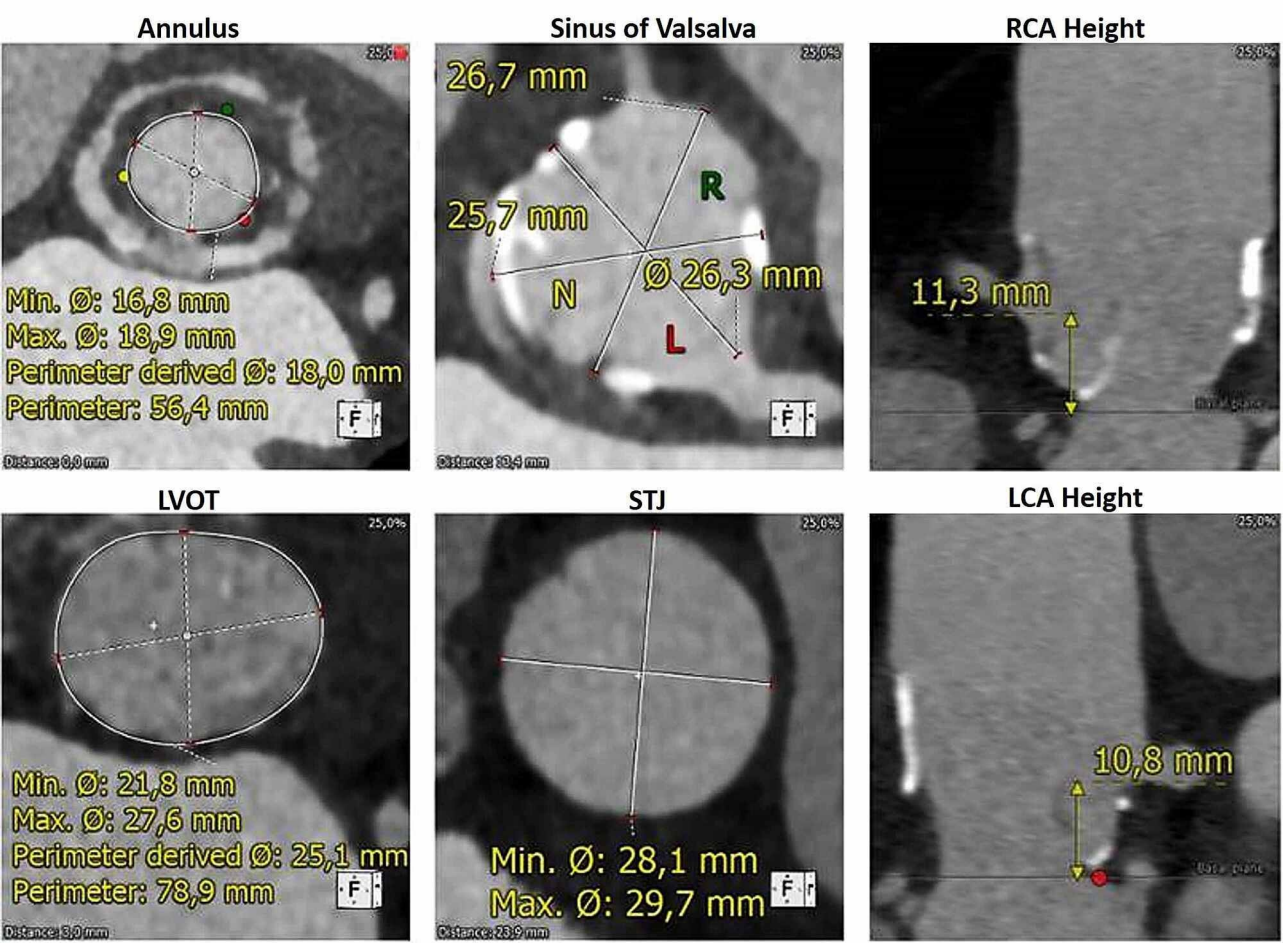
Pre-procedure CT scan LCA - left coronary artery; LVOT - left ventricle outflow tract; RCA - right coronary artery; STJ - sino-tubular junction

Transoesophageal echocardiogram showed thickness and retraction of the valve cusp positioned in the right coronary position and a prolapse of the cusp in the non-coronary position resulting in severe, eccentric, transprosthetic aortic regurgitation. There was also a mild enlargement of the left cardiac chambers (indexed left ventricular end-diastolic volume of 68ml/m2), and left ventricular systolic function was mildly depressed (left ventricle ejection fraction of 49%). Coronary artery disease was excluded by invasive angiography. A pre-procedure CT scan is shown in figure 1.

**Figure 2:**
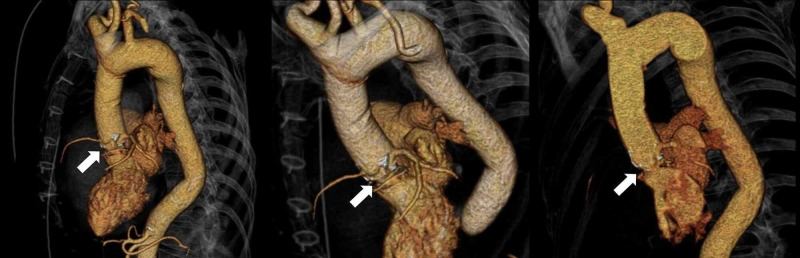
Pre-procedure three dimensional (3D) CT scan reconstruction, showing a luminal narrowing near the neo-Valsalva sinuses, which created measurement and planning difficulties

Detailed aortic annulus and aortic root measurements are shown in Table 1. Luminal narrowing, near the neo-Valsalva sinuses, introduced additional planning difficulties (Figure 2), and the measurement rendered a 23mm CoreValve as the most suitable prosthetic valve. Although not extensively, small calcium foci were present at the leaflets and the sinotubular level of the conduit. No significant calcification was present either at the annulus or left ventricular outflow tract level.

**Table 1:** Aortic annulus and aortic root measurements LCA - left coronary artery; RCA - right coronary artery

Parameter	Value
Aortic annulus	
Perimeter	56.4 mm
Area	254 mm^2^
Maximum dimension	18.9 mm
Minimum dimension	16.9 mm
LCA distance	10.8 mm
RCA distance	11.3 mm
Neo-Valsalva sinus	
Diameter	27 x 26 x 26 mm

TAVI procedure

**Figure 3:**
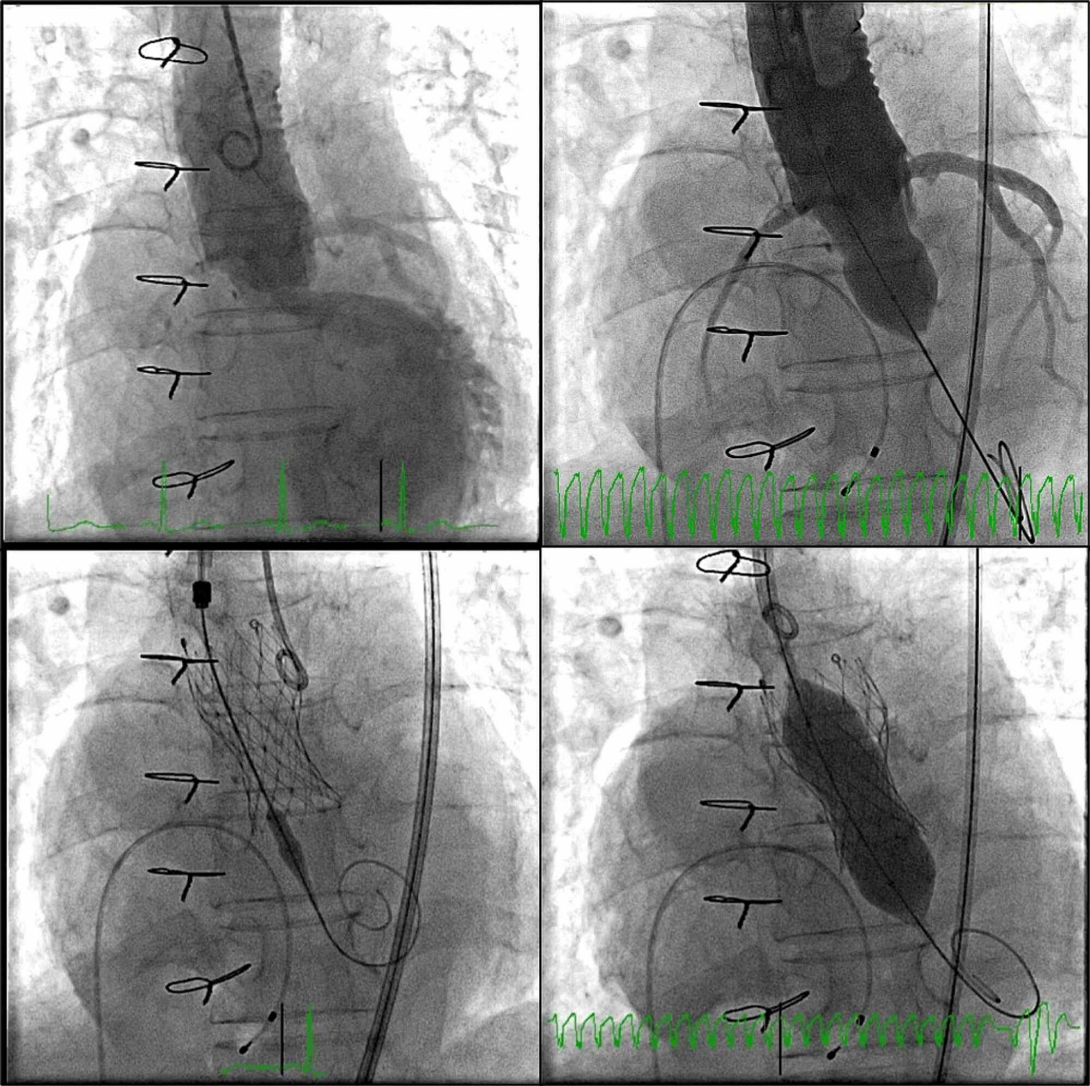
Fluoroscopic guidance during TAVI procedure; LAO 12 CRAN 5 projection was used TAVI - transcatheter aortic valve implantation; CRAN - cranial; LAO - left anterior oblique

The procedure was performed using right transfemoral access, under fluoroscopic guidance. Baseline aortography (Figure 3, left upper panel) shows severe eccentric aortic regurgitation, while it also provided coronary’s patency and height evaluation. Balloon pre-dilatation was performed with a 20mm TrueDilatation™ balloon (Figure 3, right upper panel). Pre-dilation was considered to provide additional reassurance in estimating the internal diameter of this poorly documented bioprosthesis model. It also permitted to give support to the chosen prosthesis size, which was not oversized a priori. Furthermore, adequate coronary opacification confirmed the safety of the planned implantation site. Calcium foci present at the leaflets level proved useful at this stage, helping accurate determination of the implantation depth.

A self-expandable valve prosthesis was then implanted at this stage, CoreValve Evolut R 23mm, under rapid ventricular pacing (Figure 3, left lower panel), followed by post-dilatation using the 20mm (TrueDilatation) balloon (Figure 3, right lower panel). The final angiography showed mild aortic regurgitation. There was no significant transaortic gradient by the end of the procedure. Vascular access site closure was uneventful following two suture-based pre-closure devices (ProGlide™). No electrocardiographic changes were observed in the peri-procedural period.

**Figure 4:**
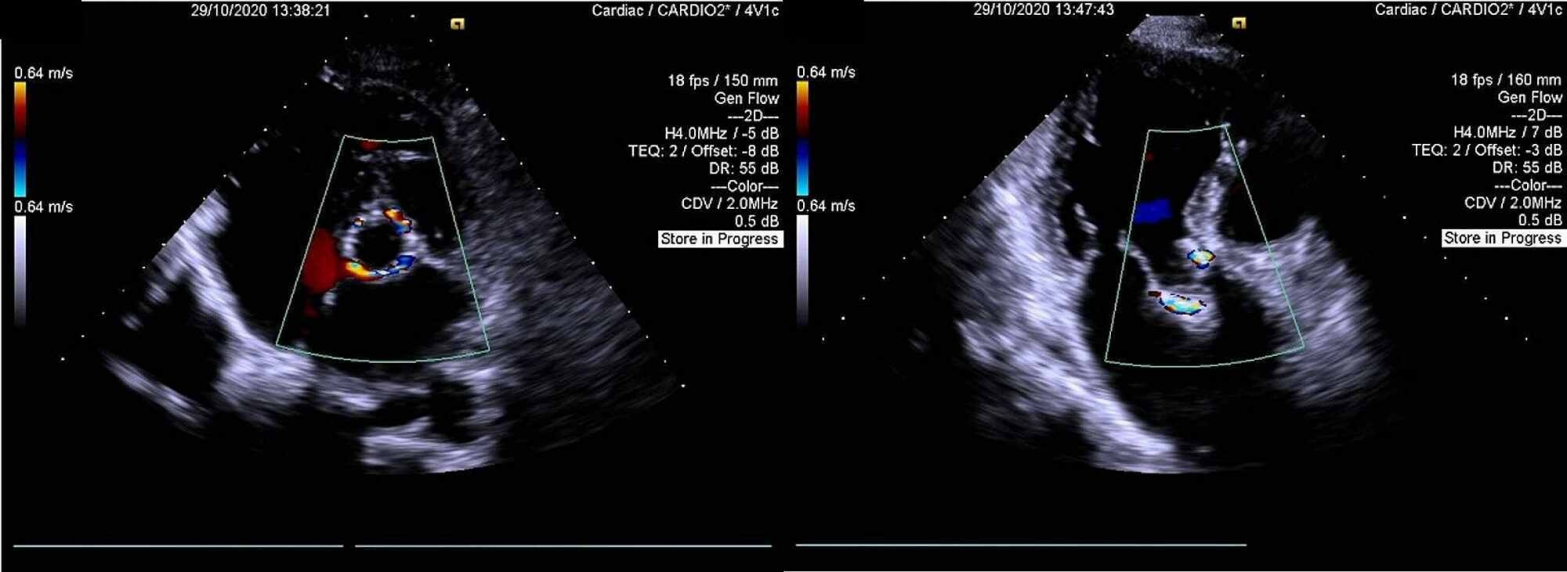
Pre-discharge transthoracic echocardiogram A parasternal short-axis view at the aortic valve level is shown in the left panel. An apical long-axis view is shown in the right panel.

Pre-discharge transthoracic echocardiogram showed biologic aortic valve prosthesis with maximum and mean gradients of 23 and 12mmHg, respectively. Mild to moderate paravalvular leak between the percutaneous aortic valve and the bioprosthetic valved conduit was visible (Figure 4).

After four days, the patient was discharged home, and a significant symptomatic improvement was documented in early follow-up (NYHA class II, two weeks following the procedure).

## Discussion

A recently published meta-analysis comprising 594 patients from six observational studies, of whom 255 underwent valve-in-valve (ViV) TAVI and 339 underwent redo surgical aortic valve replacement (SAVR), reiterated the safety and feasibility of ViV-TAVI for failed aortic bio-prosthetic valves in patients deemed to be at high risk for surgery [7]. In this analysis, no significant difference between groups was observed for procedural, 30 days, and one-year mortality rates. Valve-in-valve TAVI was associated with a lower risk of permanent pacemaker implantation with a trend toward increased risk of paravalvular leak.

Data from the Global Valve-in-Valve Registry showed that bioprosthetic valve failure might present as predominant regurgitation in up to 34% of patients, as a consequence of wear and tear, calcification, or infection [8]. ViV-TAVI was performed with the Edwards SAPIEN (Edwards Lifesciences, Irvine, USA) or the CoreValve (Medtronic, Minneapolis, USA) devices. Although stentless aortic valves represented 23% of patients in this large registry, from which 30% were homografts, the literature is scarce concerning ViV-TAVI for failed bio-prosthetic aortic conduits, predominantly due to regurgitation.

Another group reported successful treatment of a failed BioConduit with severe regurgitation, using transoesophageal echocardiographic guidance [9]. ViV TAVI after Bentall operation with a homograft was also previously reported for the treatment of severe bioprosthetic stenosis [10].

## Conclusions

This is, to the best of our knowledge, one of the first literature reports of a valve-in-valve TAVI for the treatment of late failure of a BioValsalva Vascutek conduit with severe aortic regurgitation, using the CoreValve device. The patient had an uneventful recovery in the early postoperative period and was discharged after four days. With this challenging case, the authors show the feasibility and safety of ViV-TAVI in this innovative scenario, when performed in high-volume centers and by experienced operators.
